# High manganese and nickel concentrations in human hair and well water and low calcium concentration in blood serum in a pristine area with sulphide-rich bedrock

**DOI:** 10.1007/s10653-021-01131-6

**Published:** 2021-10-26

**Authors:** Anne Kousa, Kirsti Loukola-Ruskeeniemi, Tarja Hatakka, Marjatta Kantola

**Affiliations:** 1 Environmental Solutions, Geological Survey of Finland GTK, P.O. Box 1237, 70211 Kuopio, Finland; 2 Environmental Solutions, Geological Survey of Finland GTK, P.O. Box 96, 02151 Espoo, Finland; 3 Natural Resources Institute Finland (Luke), P.O. Box 2, 00791 Helsinki, Finland

**Keywords:** Manganese, Nickel, Hair, Ground water, Well water, Medical geology, Geogenic

## Abstract

We report the trace element status of residents living in areas with naturally sulphide-rich bedrock and soil in two municipalities in Finland, Sotkamo and Kaavi. Altogether, 225 people from these sparsely populated regions participated voluntarily by providing hair and blood samples. The concentrations of calcium, zinc and copper in serum as well as selenium and cadmium in whole blood did not show correlation with those concentrations in hair samples. Calcium concentration in serum was slightly lower in the sulphide-rich areas (median value 91.4 mg/l, *n* = 103) than in the areas with adjacent sulphur-poor bedrock (median value 93.6 mg/l, *n* = 82). The concentrations of Ni and Mn in hair correlated with those in drinking water. The highest Mn and Ni concentrations in the water samples from private wells were 1620 µg/l and 51 µg/l and the highest concentrations in human hair samples 36.44 mg/kg and 12.3 mg/kg, respectively. The challenge with elevated trace element concentrations in some well waters is well documented. In northern countries (Finland, Sweden, Norway and Canada), only 10% of the population depend on private well water, and 90% have access to monitored municipal water supplies. Compared with data available from sulphide mine sites globally, the nickel and manganese concentrations in human hair samples were high in our sulphide-rich study area at Sotkamo representing the trace element status of residents under natural conditions.

## Introduction

Sulphide-rich bedrock is reflected in elevated concentrations of potentially harmful elements in soil, surface water and groundwater, as well as in organic stream and lake sediments (e.g. Gustavsson et al., 2011, 2012; Loukola-Ruskeeniemi et al., 1998, 2003). Our study focuses on sulphidic black shales which are sedimentary rocks containing > 0.5% organic, non-carbonate carbon. They host sulphide deposits and occurrences that have been mined for Cu, Ni, Zn, Mn, P, Mo, V, U, Au and platinum group elements. During weathering, acid neutralization potential varies depending on adjacent rock units, and especially on the presence of Ca-rich rocks. Hazardous environmental impacts may occur if the bedrock is in contact with surface water (Kim & Thornton, 1993; Loukola-Ruskeeniemi, 1992; Parviainen & Loukola-Ruskeeniemi, 2019; Piispanen & Nykyri, 1997). In addition to black shales, other sulphide-rich rocks may serve as natural sources of contamination for surface waters and groundwater. The bedrock in eastern and northern parts of Finland contains sulphide-rich black shales with the thickness from tens of meters to several hundreds of meters due to the folding of the black shale units during tectonic deformation (Loukola-Ruskeeniemi, 1999). Exposure to trace elements originating from anthropogenic sources or occupational exposure has been well documented (*e.g*. Chen et al., 2018; Gil et al., 2011), but the potential health effects on the population following long-term low-dose exposure to elements originating from the natural geological environment have not been well characterized (Amaral et al., 2008). Previous studies have demonstrated that long-term exposure to harmful substances, even at low levels, can cause health risks (Loukola-Ruskeeniemi et al., 1999, 2003; O’Neal and Zheng 2015). Begum et al. (2015) analysed Fe, Mn, Ni, Cr and Co concentrations in drinking water in densely populated areas of Pakistan having mafic and ultramafic bedrock. These rock types mainly contain mafic minerals such as olivine, pyroxene, serpentine and amphibole, which are rich in magnesium, calcium, iron, chromium and nickel. The inhabitants obtain their household water from groundwater and surface water sources. The authors report that the main sources of trace metals were geogenic, and mafic and ultramafic rocks were the main sources, especially for Ni and Cr (Begum et al., 2015).

Conventional and behavioural factors, as well as psychosocial ones, are risk factors for chronic diseases (Khot et al., 2003; Pänkäläinen et al., 2015; Singh-Manoux et al., 2018). However, elevated trace element concentrations in drinking water and food are also implicated, and an association has been proposed between environmental factors in well water and the incidence of chronic diseases (Kousa et al., 2006, 2012; Rubenowitz et al., 2000). An association between low Mg concentrations in groundwater and the increased risk of coronary heart disease has been reported in both Finland and Sweden (Kousa et al., 2008; Rubenowitz et al., 2000), as well as elsewhere (Jiang et al., 2016; McLeod et al., 2018). A low dietary Mg intake is associated with an increased risk of type 2 diabetes (Barbagallo & Dominguez, 2007). Arsenic, fluoride and nitrate concentrations in drinking water are suggested as potential environmental risk factors for type 1 diabetes (Chafe et al., 2018; Moltchanova et al., 2004). According to a Danish study, Mn in drinking water is associated with attention-deficit hyperactivity disorder (ADHD) (Schullehner et al., 2020).

Trace element concentrations in hair vary among the general population (Rodushkin et al. 2000). These concentrations may be associated with age, sex, hair dyes, lifestyle factors such as smoking, dietary habits and the geochemical environment. The trace element composition of scalp hair reflects long-term exposure and is not transiently disturbed by each meal, while blood and urine reflect the current or acute status of trace elements (Munakata et al., 2006; Razagui & Ghribi, 2005; Viana et al., 2014). Hair may be a more stable indicator of exposure to trace elements, while the levels in blood and urine are comparably low and strictly regulated by homeostatic mechanisms (Razagui & Ghribi, 2005). Ntihabose et al. (2018) reported that hair is a promising biomarker among children of environmental Mn exposure from drinking water, even at low levels (Ntihabose et al., 2018). Conversely, Skröder et al. (2017) reported that Mn in children’s hair does not reflect the internal dose and should therefore not be used as a biomarker of this dose (Skröder et al., 2017). The elaboration of standard procedures, validation of hair mineral analysis and delivery of detailed methodologies with well-defined reference levels are all required (Bellesteros et al., 2017; Mikulewicz et al., 2013).

Ni and Mn naturally occur in water, soil and air and can be leached from soil and bedrock (*e.g.* Lahermo et al., 2002). In water, Ni is present in its soluble form as the free ion Ni^2+^ (Haber et al., 2017). In aquatic environments, the main oxidation states of Mn are Mn(II), which is the soluble and bioavailable form, and Mn(IV), which is the insoluble form. Mn(II) predominates in the pH range 4–7. The extent to which Mn dissolves in groundwater depends on the amount of oxygen, the length of residence and, to a lesser extent, the pH of the water (ATSDR 2012; Barbeau et al., 2011; Kousa et al., 2021).

Apart from drinking water, the general population may be exposed to Ni through ambient air, food and cigarette smoke. Exposure may also occur through dermal contact with nickel-plated jewellery (ATSDR 2005a). Exposure to Ni from drinking water is usually lower than that from food (Haber et al., 2017). The absorption of Ni is low, 0.7–2.5%, when it is ingested via food or under a non-fasted state. However, the absorption is higher, 25–27%, when Ni is ingested via drinking water without food or under a fasted state (EFSA 2020). According to a report by EFSA (2020), oral exposure to Ni may be associated with eczematous flare-up reactions in the skin in nickel-sensitized persons after oral ingestion. The report also suggests that there may be an association between nickel exposure and adverse reproductive and developmental outcomes. No clear signs of neurotoxicity or cancer in humans with oral exposure have been reported (EFSA 2020). The results of an epidemiological cross-sectional study from Greece indicate that men with high Ni concentrations in hair also have high cholesterol, LDL, albumin and Ca levels, and women with high Ni in hair are more prone to high glucose and triglyceride and low sodium levels (Sazakli & Leotsinidis, 2017). Exposure to Mn may occur through food, drinking water, inhalation of air, dermal contact and the consumption of products containing an excess of Mn (ATSDR 2012). Bouchard et al. (2011) reported that the Mn levels in hair are significantly associated with the Mn intake from water, but not with the dietary intake. Exposure to Mn via drinking water can be associated with adverse neurological effects in children (ATSDR 2012; Bouchard et al., 2011). Mn in water has only been considered as a technical/aesthetic or cosmetic problem at the regulatory level. However, several more recent studies have reported that Mn derived from drinking water can be a health risk, especially for children (Bouchard et al., 2011; Oulhote et al., 2014).

Many elements, like zinc (Zn), copper (Cu) and selenium (Se), are essential micronutrients for humans. Via exposure to high oral doses, they can be toxic and cause health problems (ATSDR 2003; ATSDR 2005b; Bost et al., 2016). Exposure to high oral doses can associate gastrointestinal irritation (ATSDR 2003). An increased Cu/Zn-ratio and especially the Cu concentration in serum are associated with increased risk of incident infections in middle-aged and elderly men in Eastern Finland (Laine et al., 2020). Cadmium (Cd) exposure is associated with increased serum Cu/Zn ratios, especially in smokers (Satarug et al., 2018). Cd is a toxic element. Kidney and bone are the most sensitive targets following oral exposure and lung following inhalation exposure for Cd (ATSDR, 2008).

The role of dietary calcium in cardiovascular disease (CVD) is contradictory. The Australian study found no association between Ca intakes and risk of CVD mortality or myocardial infarction (Khan et al., 2015). Elevated serum total calcium levels were positively associated with hypertension among US adults (Sabanayagam & Shankar, 2011). Rohrman et al. (2016) observed the modest positive associations between high-normal serum calcium concentrations and risk of cardiovascular diseases, but the underlying mineral metabolism and the exact mechanisms are currently unclear.

The Geological Survey of Finland (GTK) and the University of Eastern Finland (the University of Kuopio), in co-operation with the healthcare centres of two municipalities, Kaavi and Sotkamo, in eastern Finland, initiated a study on the migration of harmful elements from sulphidic black shales to aquatic ecosystems and on the risks to human health in 1997 (Loukola-Ruskeeniemi et al., 1999). Results from comprehensive studies on trace element concentrations in the bedrock, soil, surface waters, organic stream and lake sediments, crayfish (*Astacus astacus*) and northern pike (*Esox lucius* L.) in our study areas in Kaavi and Sotkamo have been published earlier together with the data on mercury concentrations of altogether 225 human hair samples (Loukola-Ruskeeniemi et al., 2003). The present study was mainly focused on the exposure to nickel (Ni) and manganese (Mn) via the water from 82 private wells.

The natural geochemical background varies significantly across different rock types in our study areas (Gustavsson et al., 2011, 2012). A thorough understanding of the natural geochemical variation is important to protect the general population from long-term exposure. The aim of our study was to establish whether sulphide-rich bedrock is reflected under natural conditions in pristine areas in the trace element status, mainly Ni and Mn, among residents. No studies have been conducted about trace element concentrations in human hair, blood serum and whole blood samples in different geochemical areas in Finland. Finally, we compared our results with those in the vicinity of mine environments in other countries.

## Methods

### Study areas

**Fig. 1:**
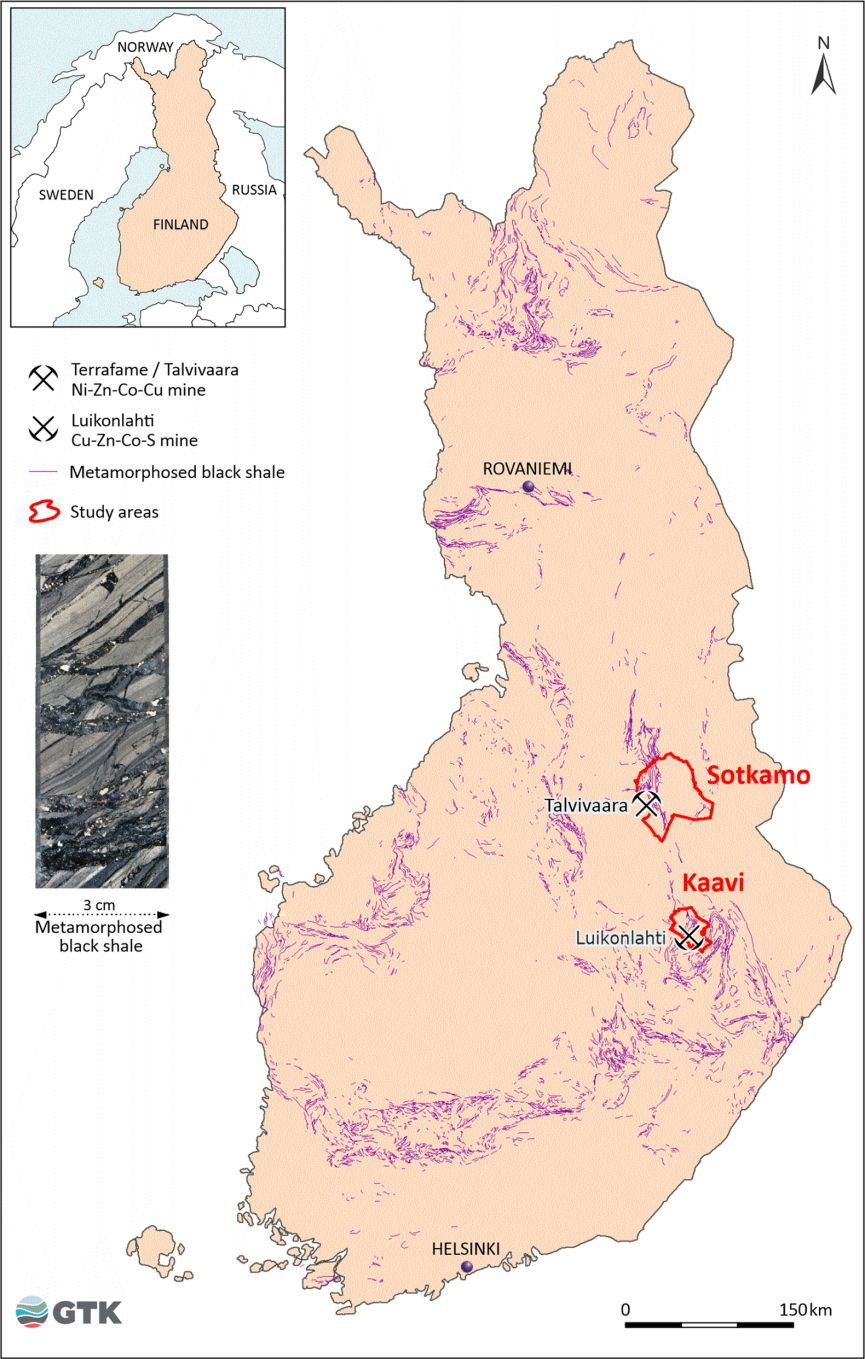
Location of the study areas and the Talvivaara Ni–Zn–Co–Cu mine (operating from year 2008) and the Luikonlahti Cu–Zn–Co–S mine (operated during 1968–1983). The distribution of Precambrian schists rich in graphite and sulphides, so-called black shales, is from the black shale database of the Geological Survey of Finland (Arkimaa et al., 1999, 2000, Loukola-Ruskeeniemi et al., 2011, and Hyvönen et al., 2013)

The data reported here were obtained from well water and human hair samples collected in 1999 from two sparsely inhabited municipalities, Kaavi and Sotkamo, in eastern Finland (Fig. 1). The study areas include units of black shales, i.e. sulphide- and graphite-rich metasedimentary rocks. The sample set was divided into black shale areas and reference areas representing other rock types with low concentrations of sulphur and graphitic carbon. The large Talvivaara Ni–Zn–Co–Cu mine currently operates in the Sotkamo black shale area (Loukola-Ruskeeniemi & Heino, 1996). Mining activities began in 2008. As our sampling campaign was carried out in 1999, the results of the present study represent background levels. A sulphide mine has operated in the Kaavi study area as well but before our sampling campaign. The Luikonlahti mine with copper, zinc, cobalt and sulphur as the main products operated between 1968 and 1983 (Fig. 1).

### Water samples

**Fig. 2:**
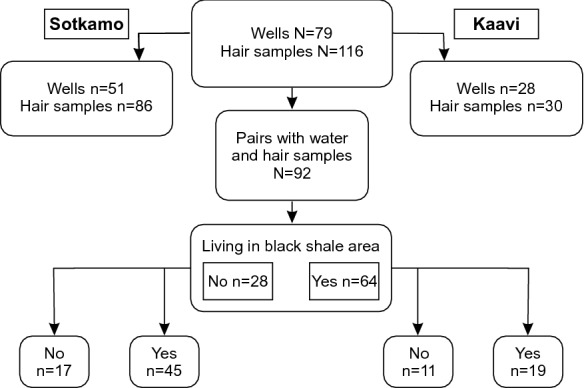
The number of hair samples from voluntary participants using private well water was 92

**Fig. 3:**
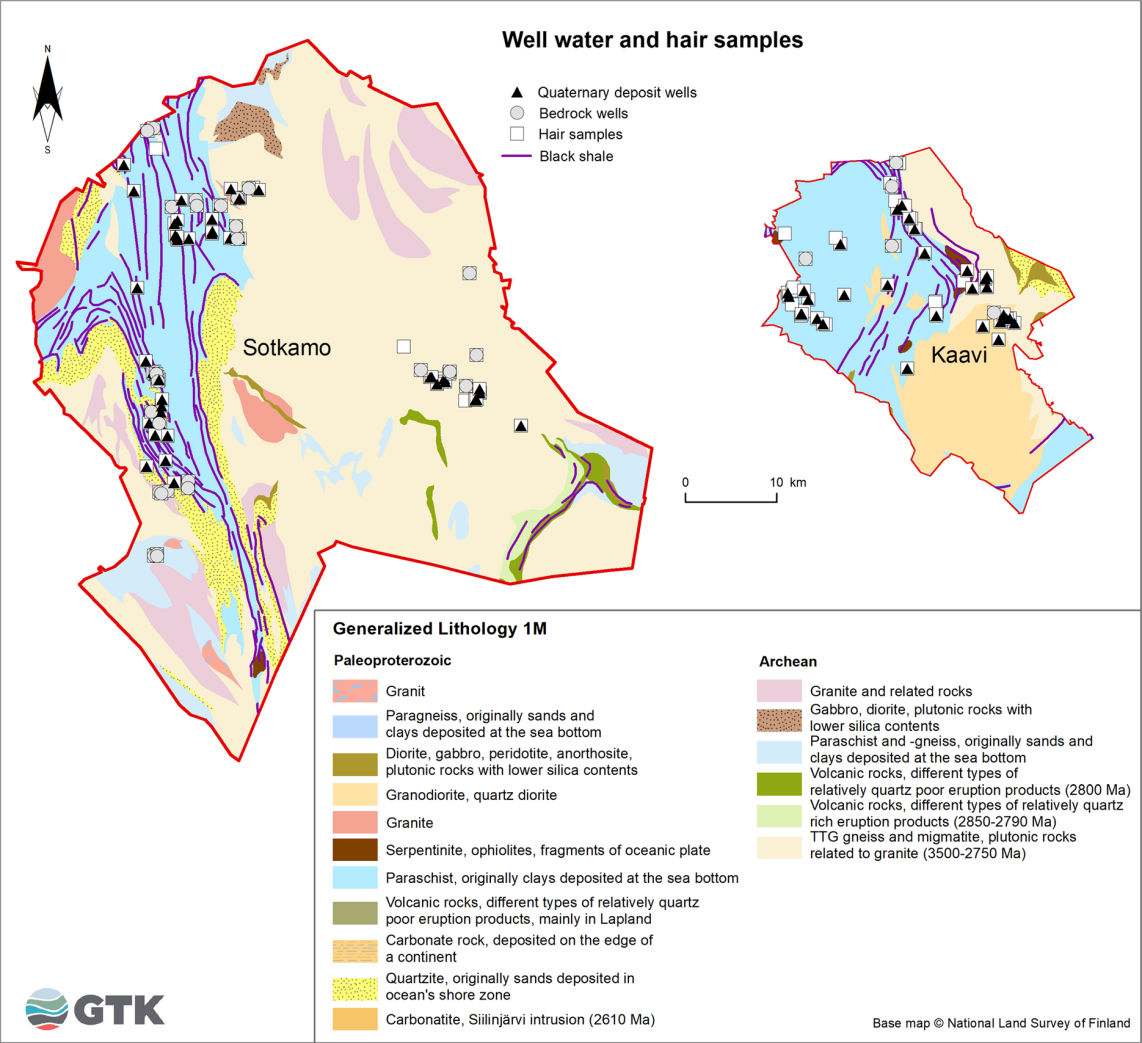
Location of private houses and voluntary residents providing both human hair and well water samples. The map of the Precambrian bedrock is based on the database of the Geological Survey of Finland (Bedrock of Finland—Digi KP)

Altogether 82 water samples, mainly private well water in active household use, were taken late in the Finnish winter between 15 and 19 March 1999. Of these, 29 samples were collected from wells drilled into Precambrian crystalline bedrock and 50 samples from springs, captured springs and dug wells representing shallow groundwater in Quaternary deposits (Fig. 2 and Fig. 3). In the Sotkamo area, 24 water samples were taken from bedrock wells and 27 from Quaternary deposit wells. In the Kaavi area, five water samples were collected from bedrock wells and 23 from Quaternary deposit wells. All samples were taken via the kitchen taps to provide water samples representing the water the residents consume daily. Here we report mainly the soluble forms of Mn and Ni in the water samples (filtered to 0.45 µm and acidified with 0.5 ml HNO_3_/100 ml water). However, the water samples were analysed in the accredited laboratory of the Geological Survey of Finland for major and trace elements using ICP-MS and ICP-AES methods and providing a long list of elements measured. Residents using municipal water were excluded, and only water samples from private wells were included in the study. The quantification limits in water were 0.02 μg/l for Mn and 0.06 µg/l for Ni. The chemical characteristics of well waters were combined with the attributes of participants from the same households. In statistical calculations, if the analysed Mn and Ni concentrations were below the limits of quantification, we used one-half of the limit of quantification as the value. The quality of sampling was assured by including 5% blank samples in each sample batch. Experienced and/or certified personnel took the water samples. Water samples were analysed in an accredited laboratory using standard or accredited methods. The accredited laboratory enforced its customary quality assurance methods. Mn and Ni concentrations in the water samples from wells drilled into crystalline bedrock and shallow groundwater (springs, captured springs and dug wells) were processed and analysed separately for the black shale and reference areas.

### Hair samples

Altogether 225 volunteers, 120 women and 105 men aged from 15 to 84 years, participated in the study. The medical staff of the municipal health centres of Sotkamo and Kaavi collected hair samples (Fig. 3). These samples were collected by cutting hair from the back of the head near the scalp using steel scissors. The samples were bound with a thread of cotton and stored in paper envelopes. Dry hair samples (3–50 mg) were treated with a mixture of supra-pure nitric acid and sulphuric acid. Sample mineralization was performed with a microwave digestion system (Loukola-Ruskeeniemi et al., 2003). Hair samples were analysed using ICP-MS/AES with a limit of quantification of 0.05 mg/kg for Mn and 0.3 mg/kg for Ni in the accredited laboratory of the Geological Survey of Finland. The number of participants for whom both the household well water and hair samples were analysed was 92 (Fig. 2). The mean age of the women and men in this study was, respectively, 57.5 ± 15.9 and 51.8 ± 14.5 years in Kaavi and 49.6 ± 17.5 and 47.7 ± 14.6 years in Sotkamo.

### Biological samples

The medical staff of the municipal health centres of Sotkamo and Kaavi collected biological samples. Samples of serum Zn, Cu, Se and samples of whole blood B-Cd and B-Se were analysed with atomic absorption spectrophotometric AAS methods at the University of Kuopio.

The study was approved by the University of Kuopio Research Ethics Committee in accordance with the Helsinki Declaration of 1975 as revised in 1983.

## Statistical analyses

The concentrations of Mn and Ni in hair and well water samples, as well as the age, sex and the length of residence of the participants were included in the statistical analyses. Only 92 participants with both household well water and hair analyses were included in the statistical model (Fig. 2). Age was classified into five groups. The normality of the distribution of elements in well water, hair and biological samples, participant age and length of residence was tested using the Kolmogorov–Smirnov test and the Shapiro–Wilk test. Element concentrations were highly skewed to the right. In the case of skewed distributions and a small number of observations, the nonparametric Mann–Whitney U-test was applied. The Kruskall–Wallis test was used for comparing Mn and Ni concentrations in hair samples according to the quartiles of Mn and Ni in well waters based on pooled data from the two municipalities. Spearman’s correlation coefficients were calculated between untransformed concentrations in hair, water and biological samples and the age and length of residence of the participants separately for the black shale and reference areas of Kaavi and Sotkamo. The correlation with smoking years (former and current smokers) was only calculated among participants of the black shale area of Sotkamo (*n* = 19). The number of smokers was too small for statistical analysis in the other subgroups. In the case of normally distributed variables, ANOVA was applied. Statistical analyses of the results were conducted using the IBM® SPSS® v.26.0.0 and 28 software and the map presentations using ArcMap 10.3 software.

## Results and discussion

### Mn and Ni concentrations in human hair compared with the values in drinking water

We compared Mn and Ni concentrations in 92 hair samples with the corresponding household well water concentrations of Mn and Ni in the black shale and reference areas of Sotkamo and Kaavi (Figs. 1,2 and 3). The general descriptive statistics for Mn and Ni concentrations in hair and well water are provided in Table 2. The Finnish national quality requirement for Ni in drinking water is below 20 µg/l. The quality recommendation for Mn, based on technical/aesthetic effects, is below 50 µg/l, and the threshold limit is 100 µg/l for small units or households using private wells (STM 1352/2015, STM 401/2001). In the water samples, 9.8% of Ni concentrations exceeded the allowable upper limit, while for Mn, 15.5% of the samples exceeded the national quality recommendation of 50 µg /l and 11.1% exceeded the threshold limit of 100 µg/l. We compared also Mn and Ni concentrations in hair of the residents in black shale areas with the reference intervals set by Biolab (2012) and the hair concentrations of residents in the vicinity of mine environments in selected countries (Table 6). In addition, we reported the concentrations of biological samples like serum Ca, Zn, Cu, Se and the whole blood Cd (B-Cd) and Se (B-Se) in the present study (Table 5).

### The sample sets

At Sotkamo, the average age of the residents participating in this study was 48.8 years in the black shale area and 49.1 years in the reference area. At Kaavi, the respective average ages were 56.0 and 53.4 years. The length of residence was on average 30.4 years in the black shale area and 22.9 years in the reference area at Sotkamo and 38.7 and 30.9 years, respectively, at Kaavi. The average duration of smoking years among the active smokers was 15.33 years in the black shale area (*n* = 6) at Sotkamo, while the participants from the reference area included only one active smoker with 30 smoking years. At Kaavi, there were no active smokers among the participants. Neither the average age nor the length of residence differs significantly between black shale and reference areas within the municipalities (*p* > 0.05).

At Sotkamo, the median Ni concentration in well water was significantly higher in the black shale area (2.79 µg/l) than in the reference area (1.17 µg/l). At Kaavi, the median concentrations of Ni (16.0 µg/l) and Mn (4.48 µg/l) in well water were higher in the black shale than in the reference area, but the difference was not statistically significant (Table 1).

**Table 1:** Mn and Ni concentrations in samples of hair and household well water from residents of black shale and reference areas in Sotkamo and Kaavi. Data are only included from households in which both hair samples and water from private well were analysed

Sotkamo	Water Mn µg/l	Hair Mn mg/kg	Water Ni µg/l	Hair Ni mg/kg
*Black shale area n* = *45*				
Median	9.18	1.30	2.79^a^	0.50
Mean ± SD	93.18 ± 335	2.23 ± 2.28	4.80 ± 6.51	0.96 ± 1.01
Range	0.28–1620	0.20–11.10	< LQ-24.20	< LQ-6.00
*Reference area n* = *17*				
Median	7,84^b^	1.37^b^	1.17^a, b^	0.77
Mean ± SD	65.50 ± 109.65	5.73 ± 9.16	1.62 ± 1.62	1.62 ± 1.99
Range	0.94–289	0.21–36.44	< LQ-6.17	< LQ-8.00
*Total n* = *62*				
Median	9.18	1.34	2.58	0.5
Mean ± SD	85.59 ± 290.53	3.19 ± 5.31	3.93 ± 5.77	1.14 ± 1.36
Range	0.28–1620	0.20–36.44	< LQ–24.20	< LQ–8.00

Kaavi	Water Mn µg/l	Hair Mn mg/kg	Water Ni µg/l	Hair Ni mg/kg
*Black shale area n* = *19*				
Median	4.48	0.91	16.00	0.89
Mean ± SD	37.64 ± 110.90	1.75 ± 2.05	12.91 ± 13.08	1.72 ± 2.70
Range	0.34–487.00	0.31–7.20	0.16–51.00	< LQ–12.31
*Reference area n* = *11*				
Median	1.77^b^	0.83^b^	6.90^b^	0.85
Mean ± SD	15.15 ± 41.89	1.56 ± 2.78	5.73 ± 2.96	1.10 ± 0.65
Range	0.42–141.00	0.25–9.86	0.18–9.15	0.43–2.58
*Total n* = *30*				
Median	1.94	0.90	6.90	0.87
Mean ± SD	29.40 ± 91.43	1.68 ± 2.31	10.28 ± 11.03	1.49 ± 2.18
Range	0.34–487.00	0.25–9.86	0.16–51.00	< LQ–12.31

SD = standard deviation, LQ = limit of quantification

^a^Mann–Whitney U-test: between black shale and reference areas of Sotkamo; Ni in water, *p* = 0.012

^b^Mann–Whitney U-test: between reference areas of Sotkamo and Kaavi; Mn in water, *p* = 0.020; Mn in hair, *p* = 0.041; Ni in water, *p* = 0.001

The concentrations of Mn and Ni in hair and water did not differ significantly between the black shale areas (Table 1). However, when comparing the reference areas, Mn concentrations in well water (7.84 µg/l) and in hair (1.37 mg/kg) were significantly higher at Sotkamo, while the Ni concentration in well water (6.90 µg/l) was higher at Kaavi (Table 1).

### Correlations between Mn and Ni concentrations in hair and water

In the black shale area at Sotkamo, statistically significant correlations were detected between the concentrations of Mn in hair and well water, Ni in hair and water, and between Ni and Mn in hair (Table 2). No significant correlations were observed between Mn and Ni concentrations in hair or well water and participant age, sex, length of residence or smoking years.

**Table 2:** Spearman correlation coefficient matrix for Mn and Ni concentrations in water and hair, age, sex, length of residence and smoking years (only in Sotkamo) in the black shale and reference areas of Sotkamo and Kaavi

Sotkamo black shale area (*n* = 45)	Mn in water	Mn in hair	Ni in water	Ni in hair
Mn in water	1			
Mn in hair	0.569^**^	1		
Ni in water	− 0.045	− 0.143	1	
Ni in hair	0.249	0.432^**^	0.337^*^	1
Age	0.042	0.012	0.083	− 0.042
Sex	− 0.044	− 0.243	0.114	0.127
Length of residence	− 0.115	− 0.022	− 0.216	− 0.172
Smoking years (n = 19)	0.199	− 0.045	0.381	− 0.046

Sotkamo reference area (*n* = 17)	Mn in water	Mn in hair	Ni in water	Ni in hair
Mn in water	1			
Mn in hair	0.450	1		
Ni in water	− 0.272	− .630^**^	1	
Ni in hair	− .682^**^	− 0.007	0.204	1
Age	0.046	− 0.193	0.461	− 0.151
Sex	0.252	0.105	0.119	0.132
Length of residence	− .515^*^	− 0.255	0.085	0.251

Kaavi Black shale area (*n* = 19)	Mn in water	Mn in hair	Ni in water	Ni in hair
Mn in water	1			
Mn in hair	0.452	1		
Ni in water	0.157	0.384	1	
Ni in hair	0.110	.577^**^	0.686^**^	1
Age	0.106	0.186	-0.142	-0.157
Sex	0.250	0.259	0.441	-0.020
Length of residence	0.053	0.091	0.084	-0.096

Kaavi reference area (*n* = 11)	Mn in water	Mn in hair	Ni in water	Ni in hair
Mn in water	1			
Mn in hair	0.856**	1		
Ni in water	0.115	0.124	1	
Ni in hair	0.366	0.409	0.494	1
Age	− 0.546	− 0.223	0.161	− 0.082
Sex	0.087	− 0.058	− 0.087	0
Length of residence	− 0.129	− 0.085	0.08	− 0.456

**Significant at the 0.01 level (2-tailed). *Significant at the 0.05 level (2-tailed).

**Fig. 4:**
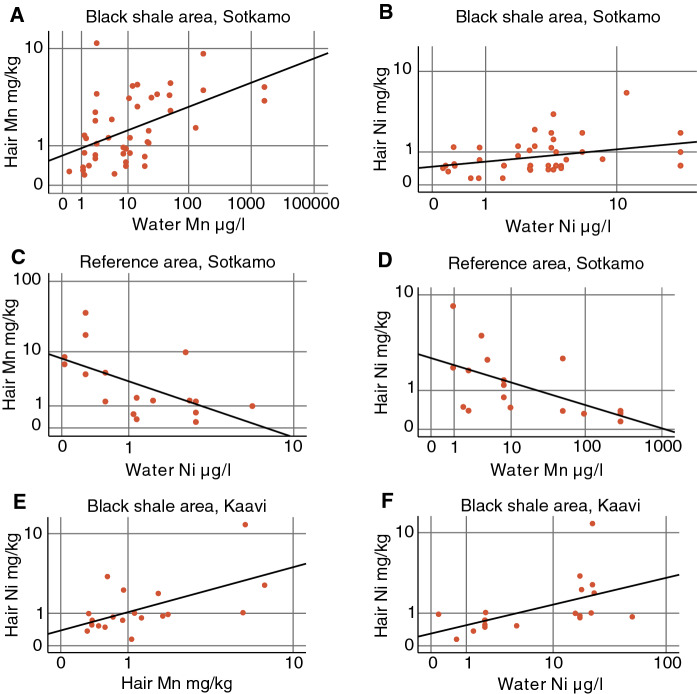
Scatter diagrams of **A** concentrations of Mn in hair and water and **B** Ni in hair and water in the black shale area at Sotkamo (n = 45), **C** concentrations of Mn in hair and Ni in water and **D** Ni in hair and Mn in water in the reference area at Sotkamo (*n* = 17), and **E** concentrations of Ni and Mn in hair, and **F** Ni in hair and water in the black shale area at Kaavi (*n* = 19). The study areas are rural and pristine, and sparsely populated

In the reference area of Sotkamo, negative correlations were observed between Ni in hair and Mn in well water, and between Ni in well water and Mn in hair (Table 2). The reason for these negative correlations is unclear. In the black shale area at Kaavi, Ni in hair correlated with Mn in hair and Ni in water (Table 2). No significant correlations were observed between other variables. In the reference area at Kaavi, Mn in hair correlated with Mn in water (*r*_s_ = 0.856). However, this correlation is tentative because of the small number of samples (*n* = 11). No other statistically significant correlations were detected (Table 2). The correlations are presented graphically in Fig. 4.

### Pooled data from the municipalities of Sotkamo and Kaavi

In the black shale areas of the pooled data set (*n* = 64), the median concentrations were 8.72 µg/l for Mn in water, 1.14 mg/kg for Mn in hair, 3.52 µg/l for Ni in water and 0.71 mg/kg for Ni in hair. In the reference areas (*n* = 28), the median concentrations were 2.81 µg/ l for Mn in water, 1.25 mg/kg for Mn in hair, 2.68 µg/l for Ni in water and 0.83 mg/kg for Ni in hair. We detected no statistically significant differences between Mn and Ni concentrations in water or in hair between the black shale and reference areas of the pooled data set. Likewise, Mn and Ni concentrations in hair did not differ significantly between men and women in the black shale or reference areas (Mn, *p* = 0.621; Ni, *p* = 0.655). Neither did we observe any difference in Mn or Ni concentrations in hair between age groups in the black shale (Mn, *p* = 0.778; Ni, *p* = 0.841) or reference areas (Mn, *p* = 0.507; Ni, *p* = 0.362).

In the pooled black shale areas, Mn in hair correlated with Mn in water (*r*_s_ = 0.578, *p* < 0.01), Ni in hair with Ni in water (*r*_s_ = 0.459, *p* < 0.01) and Mn in hair with Ni in hair (r_s_ = 0.400, *p* < 0.01). In the pooled reference areas, Mn in hair correlated positively with Mn in water (*r*_s_ = 0.640, *p* < 0.01) and negatively with Ni in water (*r*_s_ = − 0.409, *p* = 0.031).

In the pooled data for the black shale areas of the two municipalities, the median Mn concentrations in hair increased as a function of Mn concentrations in well water, being 3.94 mg/kg in the 4th quartile of Mn, > 22.40 µg/l (*p* < 0.001). Likewise, the median Ni concentrations in hair increased as a function of the Ni concentrations in well water, being 1.00 mg/kg in the 4th quartile of Ni, > 7.62 µg/l (*p* = 0.001) (Table 3).

**Table 3:** Mn and Ni concentrations in hair samples according to quartiles (Q1–Q4) of Mn and Ni concentrations in well water in the pooled data for the black shale and reference areas of the two municipalities, Sotkamo and Kaavi

Black shale area. Mn in well water (µg/l)	Mn in hair (mg/kg)		Ni in well water (µg/l)	Ni in hair (mg/kg)	
(Q1) < 2.0	Median	0.60	< 1.54	Median	0.50
	Mean	0.70		Mean	0.52
	SD	0.41		SD	0.33
	Range	0.20–1.40		Range	< LQ–1.2
	N	16		N	17
(Q2) 2.01–8.72	Median	1.01	1.55–3.52	Median	0.64
	Mean	1.91		Mean	0.80
	SD	2.66		SD	0.48
	Range	0.22–11.10		Range	0.4–2.2
	N	16		N	16
(Q3) 8.73–22.40	Median	1.14	3.53–7.62	Median	0.70
	Mean	1.74		Mean	1.01
	SD	1.44		SD	0.86
	Range	0.40–4.84		Range	0.4–3.5
	N	17		N	16
(Q4) > 22.40	Median	3.94	> 7.62	Median	1.00
	Mean	4.17		Mean	2.37
	SD	2.22		SD	3.01
	Range	0.71–9.94		Range	0.5–12.3
	N	15		N	16
Total	Median	1.14	Total	Median	0.71
	Mean	2.09		Mean	1.19
	SD	2.21		SD	1.71
	Range	0.20–11.10		Range	< LQ–12.3
	N	64		N	64
*p*-value		< 0.001^a^			0.001^b^

Reference area. Mn in well water (µg/l)	Mn in hair (mg/kg)		Ni in well water (µg/l)	Ni in hair (mg/kg)	
(Q1) < 1.67	Median	0.44	< 0.57	Median	0.47
	Mean	1.13		Mean	1.79
	SD	1.61		SD	2.66
	Range	0.25–4.70		Range	< LQ–8.0
	N	7		N	8
(Q2) 1.68–2.81	Median	0.93	0.58–2.68	Median	0.46
	Mean	0.91		Mean	1.30
	SD	0.43		SD	1.58
	Range	0.32–1.58		Range	0.3–4.3
	N	7		N	6
(Q3) 2.82–41.18	Median	1.30	2.69–5.77	Median	1.20
	Mean	2.26		Mean	1.40
	SD	3.37		SD	0.82
	Range	0.21–9.83		Range	0.6–2.6
	N	7		N	7
(Q4) > 41.18	Median	8.33	> 5.77	Median	1.10
	Mean	12.07		Mean	1.08
	SD	11.9		SD	0.46
	Range	1.37–36.44		Range	0.5–1.7
	N	7		N	7
Total	Median	1.25	Total		
	Median	0.83			
	Mean	4.09		Mean	1.41
	SD	7.54		SD	1.60
	Range	0.21–36.44		Range	< LQ–8.0
	N	28		N	28
*p*-value		0.004^a^			0.525^b^

*SD*, standard deviation, *LQ*=limit of quantification

^a^Kruskall–Wallis test between concentrations of Mn in hair according to quartiles of Mn concentrations in well water

^b^Kruskall–Wallis test between concentrations of Ni in hair according to quartiles of Ni concentrations in well water

In the pooled data for the reference areas, the median Mn concentrations in hair increased as a function of the Mn concentrations in well water, being 8.33 mg/kg in the 4th quartile of Mn, > 41.18 µg/l (*p* = 0.004). The highest Ni concentration in hair, 1.20 mg/kg, was found in the 3rd quartile of Ni in well water, 2.69–5.77 µg/l (*p* = 0.525) (Table 3). Medians of Mn in hair were higher than the upper limit of reference interval, 2.00 mg/kg for Mn, set by Biolab (2012) in the 4th quartile of Mn in well water in both, black shale and reference areas. Medians of Ni in hair did not exceed the threshold value > 1.4 mg/kg for Ni in hair (Table 3).

### Mn and Ni concentrations in Quaternary deposit wells and drilled bedrock wells

We compared Mn and Ni concentrations in hair and water among users of two different well types. In rural areas of Finland, water supplies consist of wells in Quaternary deposits (springs, captured springs and dug wells) and wells drilled into bedrock. General statistics on Mn and Ni concentrations in hair and water from both well types are presented in Table 4.

**Table 4:** Median values and ranges (2%–max) for Mn and Ni concentrations in hair and water from Quaternary deposit and bedrock wells in the black shale and reference areas of this study (pooled) and median Mn and Ni concentrations in well water for the whole of Finland (Lahermo et al., 2002)

	Black shale area		Reference area		Well water in Finland^d^	
	Quaternary deposit wells (*n* = 49)	Bedrock wells (*n* = 15)	Quaternary deposit wells (*n* = 11)	Bedrock wells (*n* = 17)	Quaternary deposit wells (*n* = 739)	Bedrock wells (*n* = 263)
Water Mn µg/l						
Median	5.07^a, b^	24.9^b^	1.77^a, c^	7.84^c^	4.36	16.3
2%–maximum	0.34–1620	0.28–172	0.42–12.90	0.94–289	0.1–5330	0.2–4140
Hair Mn mg/kg						
Median	0.94	2.03	0.83^c^	1.37^c^		
2%–maximum	0.20–11.1	0.27–9.04	0.25–9.83	0.21–36.44		
Water Ni µg/l						
Median	3.83^b^	0.65^b^	4.55^c^	1.17^c^	0.84	0.595
2%– maximum	0.32–51.0	0.15–8.15	1.10–8.87	0.03–9.15	0.06–277	< 0.06–67.50
Hair Ni mg/kg						
Median	0.85^b^	0.49^b^	0.86	0.77		
2%–maximum	0.15–12.3	0.15–2.2	0.44–4.3	0.15–8.0		

^a^Mann–Whitney U-test: difference in Mn concentrations in water between Quaternary deposit wells of black shale and reference areas, *p* = 0.018

^b^Mann–Whitney U-test: difference in Mn and Ni concentrations between Quaternary deposit and bedrock wells in the black shale areas, Mn in water *p* = 0.002, Ni in water *p* < 0.001, Ni in hair *p* = 0.007

^c^Mann–Whitney U-test: difference in Mn and Ni concentrations between Quaternary deposit and bedrock wells of the reference areas, Mn in water *p* = 0.006, Mn in hair *p* = 0.047, Ni in water *p* = 0.003

^d^Lahermo et al. (2002)

Comparing the black shale and reference areas, the median concentration of Mn was higher in water taken from both Quaternary deposit wells (*p* = 0.018) and bedrock wells (*p* > 0.05) in the black shale areas. Comparing Mn and Ni concentrations in water and hair between users of bedrock well water and Quaternary deposit well water in the black shale areas, the Mn concentration in water was significantly higher (*p* = 0.002) and that in hair nonsignificantly higher (*p* = 0.111) among the participants using bedrock well water. Higher Ni concentrations in water (*p* < 0.001) and hair Ni (*p* = 0.007) were recorded among the participants using Quaternary deposit well water.

In the reference areas, higher median values of Mn in water (*p* = 0.006) and in hair (*p* = 0.047) were detected among the users of bedrock well water, whereas Ni concentrations were higher in Quaternary deposit well water (*p* = 0.003).

In the black shale areas, the Mn concentrations in well water exceeded the Finnish threshold limit of 100 µg/l for Mn in three bedrock wells (130–172 µg/l) and three Quaternary deposit wells (487–1620 µg/l). The Ni concentrations in well water were below the national quality requirement for Ni in all bedrock wells (STM 2001, 2015). Higher Ni concentrations were recorded in nine water samples from Quaternary deposit wells in the black shale areas. In one of the samples, the Ni concentration was two and a half times higher (51 µg/l) than the national quality requirement of 20 µg/l and in eight samples slightly exceeded this regulation for Ni (concentrations 22.20–24.20 µg/l). In the reference areas, Mn concentrations in water exceeding the threshold limit were found in four bedrock wells (141–289 µg/l).

Median concentration of Mn in hair exceeded the upper limit of reference interval (Biolab 2012) only in bedrock well water users in black shale area (Table 4).

In Finland as a whole, the median Mn concentration is 4.36 µg/l in dug wells and 16.3 µg/l in bedrock wells, and the respective concentrations for Ni are 0.84 µg/l and 0.6 µg/l (Lahermo et al., 2002). In the black shale areas of this study, the Mn concentration in bedrock well water, 24.9 µg/l, was higher than the average Mn concentration in the water of bedrock wells for the whole country. In the Quaternary deposit well waters in both black shale and reference areas, the Ni concentrations were higher than those in well water in Finland on average (Table 4).

### Pooled data of biological samples from the municipalities of Sotkamo and Kaavi

Concentrations of the biological samples are presented in Table 5.

**Table 5:** Serum Zn, Cu, Se, and whole blood Cd and Se concentrations in the pooled black shale and reference areas

Black shale areas					Reference areas				
Concentration	Median	Mean SD	Range	*n*	Median	Mean SD	Range	*n*	*p-value*^*a*^
Serum Ca mg/l	91.40	91.55 ± 4.12	78.3–102.6	103	93.6	93.53 ± 3.85	85.50–103.3	82	*p* < *0.001*^*b*^
Serum Zn mg/l	0.82	0.86 ± 0.16	0.55–1.53	104	0.87	0.87 ± 0.12	0.61–1.21	82	*p* = *0.122*
Serum Cu mg/l	1.15	1.20 ± 0.26	0.79–2.28	104	1.15	1.17 ± 0.24	0.60–1.90	82	*p* = *0.610*
Serum Se µg/l	92.25	91.53 ± 10.53	69.50–113.0	104	91.50	92.26 ± 15.18	48.50–148.0	80	*p* = *0.975*
^c^B-Se µg/l	102.00	101.47 ± 10.73	76.00–125.0	97	98.50	101.12 ± 21.53	67.50–210.0	82	*p* = *0.180*
^c^B-Cd µg/l	0.25	0.37 ± 0.42	0–2.73	98	0.21	0.41 ± 0.73	0.03–5.00	82	*p* = *0.425*

^a^Mann–Whitney U-test: between black shale and reference areas (pooled data of Sotkamo and Kaavi)

^b^T-test: between black shale and reference areas (pooled data of Sotkamo and Kaavi)

^c^B-Se = whole blood Se, ^b^B-Cd = whole blood Cd

*Calcium*. Ca absorption varies inversely with dietary Ca intake. Absorption of Ca from food is about 45% at intakes of 200 mg/day, but only 15% when intakes are higher than 2,000 mg/day (Fairweather-Tait & Teucher, 2002). Fractional absorption gradually declines with age (Heaney et al., 1989). A normal range for blood calcium level is 8.6 to 10.3 mg/dl (86–103 mg/l) (UCLA Health, 2021). Median of serum Ca concentration was higher in the reference area than in the black shale area. Serum Ca did not correlate with Ca in hair (*r*_s_ = 0.100, *p* = 0.346) Ca in water (*r*_s_ = 0.143, *p* = 0.176). Ca in hair correlated with Ca in water (*r*_s_ = 0.478, *p* < 0.01).

*Zinc.* Zn is an essential micronutrient for humans. The levels of Zn in healthy adults are approximately 1 mg/l in serum (ATSDR 2005b). The concentration of serum Zn did not vary between the black shale and reference areas (*p* = 0.173). Serum Zn concentrations did not correlate with Zn in hair (*r*_s_ = − 0.011, *p* = 0.919) or well water (*r*_s_ = − 0.157, *p* = 0.134) either.

*Copper.* The general population could exposure to copper via food and drinking water, copper especially from water distribution systems. Among healthy persons, the serum Cu concentrations range up to approximately 1.5 mg/l (Barceloux DG 1999). The serum Cu concentration did not vary between the black shale and reference areas. Serum Cu did not correlate with Cu in hair (*r*_s_ = − 0.052, *p* = 0.619) or well water (*r*_s_ = − 0.133, *p* = 0.207). Cu in hair correlated with well water Cu (*r*_s_ = 0.432, *p* =  < 0.01).

*Selenium.* Se is an essential micronutrient needed by the body in small amounts. It is necessary for the function of enzymes involved in antioxidant defence, thyroid hormone metabolism and redox control of intracellular reactions (ATSDR 2003). Short-term oral exposure to high concentrations of Se may cause nausea, vomiting and diarrhoea, and long-term exposure to Se may cause selenosis, a disease characterized by hair loss (ATSDR 2003). In a study in US, the geometric mean concentration of Se in serum of all ages was approximately 125 µg/l (ATSDR 2003). In the present study, the geometric mean of serum Se was 90,92 µg/l in the black shale areas and 91.02 µg/l in the reference areas. Serum Se (*p* = 0.975) or Se in whole blood did not vary between the black shale and reference areas. Serum Se correlated with whole blood Se, *r*_s_ = 0.549, *p* =  < 0.01 and Se concentration in hair correlated slightly with Se in water, *r*_s_ = 0.260, *p* = 0.012. Serum Se and whole blood Se did not correlate with Se in hair (*r*_s_ = − 0.038, *p* = 722, *r*_s_ = − 0.041, *p* = 0.706, respectively) or Se in well water (*r*_s_ = 0.160, *p* = 0.129, *r*_s_ = 0.084, *p* = 0.442, respectively).

*Cadmium.* Kidney and bone are the most sensitive targets following oral exposure and lung following inhalation exposure. Inhalation is the predominant route of exposure to Cd for the smoking population and oral exposure for the non-smoking general population (ATSDR, 2008). The overall median blood cadmium concentration was 0.27 μg/l in men and 0.33 μg/l in women among the American adult population (Zeng et al., 2021). Smoking may cause significant increases in blood cadmium (B-Cd) levels (Jarup et al., 1998). A total number of current smokers was 26 in the present study. In the black shale areas, the median Cd concentration in blood among current smokers was 0.92 µg/l in men (*n* = 8) and 0.45 µg/l in women (*n* = 5). Among non-smokers, the medians were 0.23 µg/l in men (*n* = 39) and 0.24 µg/l in women (*n* = 46). In reference area, the median Cd concentration in blood among current smokers was 0.97 µg/l in men (*n* = 10) and 0.83 µg/l in women (*n* = 3). Among non-smokers, medians were 0.20 µg/l (*n* = 27) in men and 0.19 in women (*n* = 41). We didn’t find any correlation between Cd concentration in whole blood and Cd concentrations in hair (*r*_s_ = 0.048, *p* = 0.723) or well water (*r*_s_ = − 0.050, *p* = 0.711) in black shale areas or in reference areas (*r*_s_ = 0.094, *p* = 0.633, *r*_s_ = 0.127, *p* = 0.518), respectively. The whole blood Cd concentrations did not vary between the black shale and reference areas.

### Mn and Ni concentrations in human hair in the vicinity of mine environments in different countries

A summary of the mean values of Mn and Ni concentrations in human hair (mg/kg) in the vicinity of mining areas in different countries is presented in Table 6

**Table 6:** Mn and Ni (mg/kg) concentrations in hair in the vicinity of mining areas in the selected countries

Country	*n*	Age	Mn median	Mn mean	Mn range	Ni median	Ni mean	Ni range
*Portugal*^*a*^	39	15–46						
S.Domingos	20			2.61 ± 1.04	0.022–19.62			
Corte do Pinto	9			2.99 ± 1.07	0.056–12.48			
Santana de Cambas	10			10.81 ± 3.42	0.023–35.48			
*Zambia*^*b*^	na	11–40						
Mugala				16.3 ± 2.3	3.9–44.0		1.3 ± 0.23	0.21–6.06
*Italy*^*c*^		11–14						
Iglesias and Sant'Antioco, Sardinia			0.31			0.22		
*Russia*^*d*^	128	7–14						
Ishmurzino	33		1.09	1.43 ± 0.51	0.61–3.43	0.18	0.24 ± 0.07	0.15–0.50
Semenovsk	30		1.07	1.21 ± 0.34	0.31–2.65	0.28	0.30 ± 0.06	0.17–0.47
Tubinsk	65		2.34	2.38 ± 0.20	1.64–3.21	0.27	0.45 ± 0.17	0.11–2.06
*Finland*^*e*^	64	15–85						
Sotkamo black shale	45	15–84	1.30	2.23 ± 2.28	0.20–11.10	0.50	0.96 ± 1.01	< LQ-6.00
Kaavi black shale	19	28–85	0.91	1.75 ± 2.05	0.31–7.20	0.89	1.72 ± 2.70	< LQ-12.31
*Reference interval*^*f*^	Mn 0.20–2.00, Ni < 1.4							

^a^Pereira et al. 2004

^b^Nakaona et al. 2020

^c^Tamburo et al. 2016

^d^Semenova et al. 2018

^e^present study

^f^Biolab Medical Unit 2012

The sampling area of a Portuguese study consists of the neighbourhood of the S.Domingos mine, an abandoned cupric pyrite mine located in the Southeast Alentejo, and two nearby villages, Corte do Pinto, located north of the mine, and Santana de Cambas, south of the mine (Pereira et al., 2004). The mean concentrations of Mn in hair are in line with those in the Sotkamo black shale area except for those among residents of the Santana de Cambas where Mn concentrations are higher (Table 6). Maximum concentrations of Mn are much higher than those in our study. The highest average concentration of Mn in hair is recorded in inhabitants from the Corte do Pinto and Santana de Cambas localities, where the highest concentration of Mn is recorded in the soil (Pereira et al., 2004). The results suggest that the population of the S.Domingos mine area and neighbour localities may be exposed to Mn. There is no evidence that water supplies are a potential source of heavy metals. The group that reported to consume only bottled water present the highest concentrations of Mn in the scalp hair (Pereira et al., 2016).

The study area of Zambia, Mugala village, is situated in Kitwe West on the Copperbelt Province of Zambia. It is located near the Mopani tailings dump site. Consumption of vegetables grown in the contaminated areas of the Copperbelt and the large-scale mining operations have affected the health of residents (Nakaona et al., 2020). The mean and maximum concentrations of Mn in hair are higher while Ni concentrations are lower than those in our study. In the Zambian study, the high Ni dietary intake from drinking water seems to accumulate in toenails while Mn is highly accumulated in hair (Nakaona et al., 2020).

In the Italian study, Iglesias and Sant'Antioco, the long-lasting polymetallic mining areas, locate in southwestern Sardinia. The outcrops of sulphide and oxide ores characterize the district with high concentrations of metals and metalloids (Tamburo et al., 2016). The median concentrations of Mn and Ni in hair are lower than those in the black shale areas in Finland. The authors suggest that Ni in hair shows 2.5-fold higher concentration in female hair (Tamburo et al., 2016).

The Russian study area is in the rural settlements on the territory of the Baymaksky District of the Republic of Bashkortostan (Russia) located in the vicinity of abandoned mines (Semenova et al., 2018). The median concentrations of Mn are slightly higher in the Sotkamo black shale area than those reported in the Russian study except for hair samples from Tubinsk where Mn concentrations are higher than in our study. The median and maximum values of Ni are higher in our study compared to those reported in the Russian study (Table 6).

*Mean* values of the Mn concentrations in hair exceed the upper limit of reference interval 2.0 µg/kg (Biolab Medical Unit, 2012) in all study areas listed in Table 6 except in Ishmurzino and Semenovsk in Russia and the black shale area in Kaavi. In Tubinsk, the *median* concentration of Mn exceeds the maximum reference value. *Maximum* of Mn concentrations exceed 2.0 µg/l in all available hair samples. All *median* values of Ni in hair are below the threshold value of 1.4 µg/kg set by Biolab Medical Unit for Ni while *mean* value of Ni exceeded the threshold value in the black shale area of Kaavi. *Maximum* Ni concentrations exceed the threshold value for Ni in Zambia, Tubinsk (Russia) and in the black shale areas of Sotkamo and Kaavi (Table 6).

In a Swedish study, the median Mn and Ni concentrations in hair are reported to be 0.35 mg/kg and 0.29 mg/kg, and the maximum values are 2.41 mg/kg and 1.60 mg/kg, respectively (Rodushkin et al. 2000). At Sotkamo and Kaavi, the median and maximum concentrations of Mn and Ni hair in the black shale and reference areas were higher than the concentrations reported in the Swedish study (Rodushkin et al., 1999). The chemical characteristics of the Paleoproterozoic bedrock underlying the Swedish study areas differ from the bedrock of our study areas in Finland (Koistinen et al., 2001), while the Quaternary deposits are quite similar (Salminen et al., 2005). In our study, Mn and Ni concentrations in hair were consistent with the ranges from different countries reported by Rodushkin et al. (2000).

In a Russian study, the median Mn concentration in hair is significantly higher in men than in women (Skalny et al., 2015), while in a Greek data set, Ni concentrations in hair are higher in women than men (Sazakli & Leotsinidis, 2017). In the present data, we found no marked differences in concentrations of Mn or Ni in hair in relation to age or between men and women. In comparison with the median Mn concentrations in our study, the authors of a study among a population living in the European part of Russia report lower Mn concentrations in hair, the median being 0.52 mg/kg (Skalny et al., 2015). One potential explanation is that the underlying bedrock of the European part of Russia is mostly different from the bedrock in the Palaeoproterozoic Fennoscandian Shield in our study areas (Koistinen et al., 2001). Age, sex, length of residence and smoking years did not correlate with Mn or Ni concentrations in hair among the participants in our study.

### Drinking water from private wells

Sulphide-rich rocks are potential natural sources of environmental exposure to trace elements via well water. In the black shale areas, the elevated Mn concentration in bedrock well water results from the long retention time of water passing through bedrock fractures as well as from the prevailing reducing conditions in these wells (Korkka-Niemi, 2001). In Finland, well waters from Quaternary deposits are slightly more acidic than those from deep bedrock wells resulting in the dissolution of metals, including Ni, into the water. In addition, old dug wells and household plumbing systems may promote Ni dissolution into water (Lahermo et al., 2002). The Mn and Ni concentrations in water were on average slightly higher in both well types in the black shale areas compared to the average concentrations for thousands of private wells the Geological Survey of Finland has studied in Finland. In the reference areas, the Mn concentrations in water were lower, while the Ni concentrations in water were higher than the average for the whole country (Lahermo et al., 2002).

In Finland and other Nordic countries, over 90% of households are within the municipal water supply system (Gunnarsdottir et al., 2020). Approximately 10% of the total Finnish population uses water for drinking, washing and irrigation from private single-household wells. An increasing number of summer houses increase the amount of private well water users during holiday seasons (Lahermo et al., 2002).

### Mining activities in a naturally sulphide-rich site

Our results provided background data for the evaluation of the impact of mining activities to human health. Mining of the large black-shale-hosted Ni-Zn-Cu-Co deposit at Talvivaara started in 2008 in our sulphur-rich study area in Sotkamo. Our results represent geochemical background levels since we carried out sampling already in 1999, well before the mining activities began. We recommend geochemical background studies and the monitoring of the quality of groundwater and surface waters before starting mining operations since in most cases the bedrock and soil contain potentially harmful elements and compounds already under natural conditions in a sulphide mine site. The risks to groundwater, surface waters and human health due to civil engineering and other anthropogenic actions are higher than in areas with naturally sulphide-poor bedrock and soil.

## Conclusions

The results of our study indicated that natural exposure to elevated concentrations of Mn and Ni in drinking water can be detected in human hair samples. The average levels of Mn and Ni in well water in our study areas in Sotkamo and Kaavi, eastern Finland, were below the guidelines set by the Finnish national regulations. The biological samples, the serum Ca, Zn, Cu, Se and B-Se and B-Cd concentrations, did not associate with the concentrations of those elements in well water or human hair. The chemical composition of the biological samples studied did not vary between sulphidic and sulphur-poor areas except for serum Ca which was slightly lower in sulphidic areas. The limitation of the present study was the small number of residents due to the low population density in the study areas in rural Finland. In spite of this challenge, our study described the effects of sulphur-rich bedrock and soil to human population under natural conditions. Sulphur-rich bedrock areas are encountered in highly populated countries like China, South Korea and some African countries (*e.g.* Parviainen & Loukola-Ruskeeniemi, 2019).

## Data Availability

The data that support the findings of this study are available on request from Dr. Kirsti Loukola-Ruskeeniemi.
